# Effects of psychosocial characteristics on cognitive function in middle-aged and older adults: Focusing on change by year using the Korean Longitudinal Study of Aging panel data (2014–2018)

**DOI:** 10.1097/MD.0000000000038637

**Published:** 2024-06-28

**Authors:** Ji-Young Park, Hye-Sun Jung

**Affiliations:** a Department of Health, The Catholic University of Korea, Seoul, Korea; b Department of Preventive Medicine, College of Medicine, The Catholic University of Korea, Seoul, Korea.

## Abstract

Old age is associated with a higher risk of dementia. Psychosocial characteristics frequently affect cognitive function; however, the exact mechanism underlying the effect of psychosocial factors on cognitive function is unclear. Therefore, this study aimed to investigate the effects of psychosocial characteristics on cognitive function. The participants comprised 4809 middle-aged and older (years 50+) adults. The analysis used data from the Korean Longitudinal Study of Aging from 2014 to 2018. The effects of neighborhood interaction, depression, life satisfaction, and economic activity on cognitive function were examined, and a linear mixed model analysis was performed to assess the change in cognitive function by year. A statistically significant association was found between neighborhood interaction and time. Additionally, cognitive function decreased in the presence of depression and with time. In men, significant interactions were found between depression and time and between economic activity and time. In women, significant interactions were found between life satisfaction and time. The findings indicate that since active neighborhood interaction positively affects cognitive function, it is necessary to develop various community-wide social activity programs for middle-aged and older adults. As depression is a risk factor for cognitive impairment, it is crucial to prevent cognitive decline through continuous management of depression. Given the positive effects of economic activity on cognitive function in men, it is essential to expand infrastructure to sustain economic activity by developing educational programs and creating job opportunities for middle-aged and older men.

## 1. Introduction

Korea’s population is rapidly aging due to decreasing fertility and increasing life expectancy at birth.^[1]^ In 2018, life expectancy at birth was 82.7 years, marking an increase of more than 20 years from the 62.3 years in 1970 and surpassing the Organization for Economic Co-operation and Development’s average age by 1.7 and 2.4 years in men and women, respectively.^[2,3]^ Since Korea entered a changing and aging society in 2000, its population has been aging at an unprecedented pace. The proportion of the elderly population (≥65 years) is projected to increase from 15.7% (8.1 million) in 2020 to 46.5% (18.7 million) in 2067, and the number of old-old (75+) Koreans is expected to peak at 11.8 million in 2060.^[4]^

Notably, this surge in the elderly population comes with a drastic increase in the number of patients with dementia: the prevalence of dementia in Korea was estimated at 10.2% (n = 750 000) in 2018 and is projected to reach the mark of 1 million in 2024 (10.4%) and 3 million in 2050 (16.1%).^[5]^ Therefore, with the rapidly aging population, there is a growing interest in mild cognitive impairment, i.e., the pre-dementia stage, with its prevalence estimated at 22.5%, as of 2017.^[6]^ This suggests that dementia and mild cognitive impairment will likely progress with cognitive decline over the years.^[7]^

Cognitive decline in old age reduces an individual’s ability to perform activities of daily living, results in a deterioration in the quality of life, and reduces the ability to lead an independent life, thereby increasing the social burden of care. Although the cognitive function of middle-aged and older adults may be affected by conditions such as dementia, previous studies on cognitive function have identified sex, education, age, and meeting with friends as factors associated with cognitive impairment.^[8]^ As it is known that psychosocial characteristics affect cognitive function in middle-aged and older adults, intensive research has been underway to examine social relationships, including social networks and interactions, in middle-aged and older adults.^[9]^

A previous study including middle-aged and older adults revealed that social relationships affect cognitive function, health, and psychological well-being.^[10]^ However, middle-aged and older people experience fewer social interactions and relationships with close people over the years due to retirement, children’s marriages, bereavement, and changes in their social environment.^[11]^ Conversely, they can also maintain social relationships through leisure activities, which foster cognitive function,^[12]^ and active participation in social activities also positively affects cognitive function.^[13]^

Cognitive function, which is governed by the cerebral cortex, involves the cognitive and sensory processes responsible for thinking, perceiving, and remembering objects.^[14]^ Middle-aged and older adults undergo a slow and gradual decline in cognitive function through a normal aging process.^[15]^ In addition, cognitive decline is associated with various causal factors, such as diet, socioeconomic activity, smoking, drinking, genetics, and lifestyle.^[16]^ Since cognitive decline can lead to dementia,^[17]^ it is necessary to explore methods to prevent mild cognitive impairment and dementia in middle-aged to older adults.

Although several studies have been conducted on the cognitive function of older adults, mostly on factors influencing cognitive function in middle-aged and older adults^[17]^ or on changes in cognitive function associated with living alone,^[18]^ only a few studies have reported on changes in cognitive function from multiple perspectives in middle-aged and older adults. Notably, middle-aged and older adults often find themselves at a stage of the family lifecycle wherein their children grow up and leave home and their spouses prepare for retirement, or they may experience bereavement.^[8]^ Moreover, various diseases contribute to cognitive decline. According to previous studies, middle-aged and elderly people who have experienced strokes or peripheral artery disease have a higher rate of cognitive impairment than those who have not.^[19]^ Additionally, other studies have reported that hypertension and obesity have adverse effects on cognitive function.^[20]^

As psychosocial characteristics affect cognitive function and health deterioration in middle-aged and older adults as an aging process,^[21]^ studies have been conducted to better understand how they affect cognitive function. For example, Park et al^[22]^ found a statistically significant effect of socioeconomic status (e.g., education, income, and economic activity) on cognitive function and noted that while depression negatively affects cognitive function in middle-aged and older adults, higher income can act as a buffer against cognitive decline. Additionally, Ryu et al^[23]^ demonstrated that the frequency of interacting with family and relatives, as well as with friends and neighbors, was positively correlated with cognitive function in middle-aged and older adults at a statistically significant level. This suggests that the more active the social interaction, the higher the cognitive function of middle-aged and older adults. Son et al^[24]^ found that the lower the level of depression, the higher the cognitive function, and Kim et al^[25]^ reported that the cognitive function of middle-aged and older adults was positively correlated with life satisfaction. Based on these findings, income, depression, and social relationships are factors influencing cognitive function in middle-aged and older adults.

As examined above, psychosocial characteristics frequently affect cognitive function. Neighborhood interaction can contribute to maintaining cognitive function in middle-aged and older adults because it fosters communication, participation in various social interactions, and mobilization of psychological, cognitive, and emotional abilities.^[26]^ Furthermore, meeting and talking with others can stimulate mental activities in middle-aged and older adults and prevent cognitive decline.^[27,28]^ Neighborhood interaction can reduce depression while establishing a social network, ultimately contributing to maintaining cognitive function by enhancing life satisfaction through participation in various social activities.^[29]^ Economic activity also helps maintain cognitive function in middle-aged and older adults.^[30]^ Therefore, it is crucial to determine how psychosocial factors affect cognitive function.

This study aimed to examine the effects of psychosocial characteristics (neighborhood interaction, depression, life satisfaction, and economic activity) on cognitive function after correcting for variables that can affect cognitive function, including age, education, marital status, household income, religion, and physical function. This study used the Korean Longitudinal Study of Aging (KLoSA) panel data to analyze the effects of psychosocial characteristics on cognitive function.

## 2. Methods

### 2.1. Analysis of data

This study analyzed the effects of psychosocial characteristics on cognitive decline over the years using the data from the fifth, sixth, and eighth waves of the KLoSA. The KLoSA is conducted by the Korea Employment Information Service on 10,254 middle-aged and older adults (≥45 years) living in Korea, excluding those living in Jeju Island, by computer-aided personal interviewing.

### 2.2. Research participants

Therefore, the target population of this study included middle-aged and older adults whose age range was generally considered to be 40 to 60 years.^[31]^ The South Korean Welfare of Senior Citizens Act defines senior citizens as those aged 65 and over. In this study, middle-aged and older adults were defined as those aged 50 years and over.

The total number of participants in this study was 4809 (1973 males and 2836 females), and the data were publicly available. The selection criteria were as follows: men and women, 50 + years; surveyed in all 3 waves of KLoSA (the fifth, sixth, and eighth waves); the availability of complete data containing information on the variables of this study was a prerequisite.

### 2.3. Data collection

The KLoSA panel data (raw data from the fifth, sixth, and eighth waves, questionnaire, and codebook) were downloaded for research purposes after signing up as a member at the portal of the Korea Employment Information Service Employment Survey (http://survey.keis.or.kr), disclosing the researcher’s personal information and explaining the purpose of use, and agreeing to comply with the management regulations. This study was conducted in accordance with the Declaration of Helsinki and approved by the Institutional Review Board of The Catholic University of Korea (IRB no. MC21Z AS10081).

### 2.4. Study variables

#### 2.4.1. General characteristics

Among the participants’ general characteristics, 5 items, namely, age, education, marital status, household income, and religion, were selected as variables.

#### 2.4.2. Physical characteristics

Six items, i.e., health condition, instrumental activities of daily living (IADLs), cerebrovascular disease, drinking, smoking, and workout, were used as variables among the participants’ physical characteristics.

Health condition was classified into good and poor groups by dichotomizing the very good, fairly good, and neutral groups into the good group and the poor and very poor groups into the poor group.

The IADLs were quantified using the IADL scale provided by the KLoSA. This comprises 10 items that measure the degree of dependence on others in performing the following activities of daily living: personal grooming, performing basic household chores, preparing meals, doing laundry, going out a short distance, using transportation, shopping, managing money, making and taking telephone calls, and taking medications. In the IADL scale, the responses to the 10 assessment items were scored by converting the response options of each item into a dummy variable. In this study, the responses were dichotomized into “restricted” (when help is needed for performing one or more IADLs) and “not restricted” (when no help is needed).

Cerebrovascular disease status was categorized into “yes” (when diagnosed with one or more symptoms of stroke, cerebral hemorrhage, or cerebral infarction) and “no” (absence of symptoms). Furthermore, drinking and smoking were categorized into “yes” and “no.” The workout was classified as “yes” if exercise was performed at least once a week and “no” if not.

#### 2.4.3. Psychosocial characteristics

Regarding psychosocial characteristics, the following 4 variables were used: neighborhood interaction, depression, life satisfaction, and economic activity. Various psychosocial characteristics, such as neighborhood interaction and life satisfaction, are important determinants of quality of life.^[32]^

The KLoSA defines neighborhood interaction as participation in any of the following activities: religious gatherings, social gatherings (e.g., rotational financing group meetings and senior welfare center meetings), leisure/cultural/sports groups, dedicated organizations (e.g., senior colleges), alumni/homecoming meetings/extended family meetings, volunteer work, and political parties/civic groups/interest groups.

Depression was measured using the Center for Epidemiologic Studies Depression Scale. Ten items asking for changes in feelings, attitudes, emotions, and behavior over the past week were used. Responses to each item were rated on a 4-point Likert scale: 0 = rarely or never (> 1 day), 1 = sometimes (1–2 days), 2 = often (3–4 days), and 3 = always (5–7 days), with a higher score indicating higher severity of depression. A total score of 0 to 3 was classified as normal, and a score of ≥4 was defined as depression.^[33]^

In the KLoSA, life satisfaction was assessed by the overall quality of life (happiness) on a 0-to-100 scale at 10-point intervals excluding 0, with a total score closer to 100 indicating higher life satisfaction. Those with total scores >50 were classified as “satisfied” and ≤50 as “not satisfied.”

Economic activity was classified into “yes” (presence of earnings) and “no” (absence of earnings).

#### 2.4.4. Cognitive function

Cognitive function was measured using the Korean version of the Korean-Mini Mental State Examination (K-MMSE) used by the KLoSA.^[34]^ The K-MMSE comprises 19 items, including orientation, memory, attention and calculation, recognizing personal belongings, language, and the ability to follow simple commands. The total score ranged from 0 to 30, with a higher score indicating better cognitive function. A K-MMSE score of 23 or lower indicated impaired cognitive function, and a score of 24 or higher showed normal cognitive function.

### 2.5. Data analysis

Data analysis was performed using Statistical Package for the Social Sciences (SPSS) version 25.0 (SPSS Inc., Chicago). First, frequency analysis and descriptive statistics were conducted to determine the participants’ general characteristics and cognitive function.

Second, a chi-square test was conducted to measure the differences in cognitive function according to sex, neighborhood interaction, depression, life satisfaction, and economic activity over the years.

Third, the effects of psychosocial characteristics (neighborhood interaction, depression, life satisfaction, and economic activity) on cognitive function were investigated by sex. In addition, a linear mixed model analysis was performed to examine the differences in the change in cognitive function by year, according to psychosocial characteristics.

## 3. Results

### 3.1. Participants’ characteristics and cognitive function level

Table 1 shows the participants’ general characteristics and cognitive function level in 2014 (fifth KLoSA wave). The characteristics of the male participants were as follows: the mean age was 70.76 years; 38.2% had graduated from high school; 90.9% had a spouse; 49.4% had a household income of 25 million won or more; 31.0% were religious; 46.1% perceived their health condition as “good”; 11.4% had limited IADL status; cerebrovascular disease was diagnosed in 6.3%; 53.1% consumed alcohol; 18.7% smoked; 38.4% engaged in a workout; 91.2% interacted with neighbors; 37.4% had depressive symptoms; 74.5% were satisfied with their lives; 48.0% were engaged in economic activities; impaired cognitive function was found in 22.7%.

**Table 1:** Characteristics and cognitive function of participants.

Characteristics	Classification	Men	Women
		N (%)	N (%)
Age (years)	50–59	511 (25.9)	798 (28.1)
	60–69	731 (37.1)	929 (32.8)
	70–79	560 (28.4)	823 (29.0)
	≥ 80	171 (8.7)	286 (10.1)
	M ± SD	70.76 ± 8.88	70.72 ± 9.31
	Elementary school	Education	1489 (52.5)
	Middle school		502 (17.7)
	College	754 (38.2)	710 (25.0)
	College and above	367 (18.6)	135 (4.8)
Marital status	With spouse	1793 (90.9)	1830 (64.5)
	Without spouse	180 (9.1)	1006 (35.5)
Household income	<KRW 10 million	320 (16.2)	708 (25.0)
	KRW 10–24.99 million	678 (34.4)	972 (34.3)
	≥KRW 25 million	975 (49.4)	1156 (40.8)
Religion	Religious	611 (31.0)	1218 (42.9)
	Non-religious	1362 (69.0)	1618 (57.1)
Health condition	Good	910 (46.1)	1039 (36.6)
	Poor	1063 (53.9)	1797 (63.4)
Instrumental activities of daily living	Restricted	225 (11.4)	251 (8.9)
	Not restricted	1748 (88.6)	2585 (91.1)
Cerebrovascular disease	Yes	125 (6.3)	146 (5.1)
	No	1848 (93.7)	2690 (94.9)
Drinking	Yes	1047 (53.1)	437 (15.4)
	No	926 (46.9)	2399 (84.6)
Smoking	Yes	369 (18.7)	42 (1.5)
	No	1604 (81.3)	2794 (98.5)
Workout	Yes	757 (38.4)	860 (30.3)
	No	1216 (61.6)	1976 (69.7)
Neighborhood interaction	Yes	1799 (91.2)	2594 (91.5)
	No	174 (8.8)	242 (8.5)
Depression	Yes	737 (37.4)	1247 (44.0)
	No	1236 (62.6)	1589 (56.0)
Life satisfaction	Satisfied	1469 (74.5)	1929 (68.0)
	Not satisfied	504 (25.5)	907 (32.0)
Economic activity	Yes	947 (48.0)	738 (26.0)
	No	1026 (52.0)	2098 (74.0)
Cognitive function	Normal	1526 (77.3)	1871 (66.0)
	Cognitive decline	447 (22.7)	965 (34.0)

KRW = Korean Republic won, M = mean, SD = standard deviation.

The characteristics of the female participants were as follows: the mean age was 70.72 years; 52.5% received primary education or lower; 64.5% had a spouse; 40.8% had a household income of 25 million won or more; 42.9% were religious; 36.6% perceived their health condition as “good”; 8.9% had limited IADL status; cerebrovascular disease was diagnosed in 5.1%; 15.4% consumed alcohol; 1.5% smoked; 30.3% engaged in a workout; 91.5% interacted with neighbors; 44.0% had depressive symptoms; 68.0% were satisfied with their lives; 26.0% were engaged in economic activities; impaired cognitive function was found in 34.0%.

### 3.2. Cognitive function according to participants’ characteristics

#### 3.2.1. Male participants

Analysis of the 2014 data revealed significant differences in cognitive function according to age, education, marital status, household income, health condition, IADL, cerebrovascular disease, drinking, workout, neighborhood interaction, depression, life satisfaction, and economic activity (*P < *.001), and no significant differences according to religion and smoking (*P > *.05).

Analysis of 2016 data revealed significant differences in cognitive function according to age, education, marital status, household income, health condition, IADL, cerebrovascular disease, drinking, smoking, workout, neighborhood interaction, depression, life satisfaction, and economic activity (*P < *.01), but no significant difference according to religion (*P > *.05).

Analysis of 2018 data revealed significant differences in cognitive function according to age, education, marital status, household income, religion, health condition, IADL, cerebrovascular disease, drinking, workout, neighborhood interaction, depression, life satisfaction, and economic activity (*P* < .01), but no significant difference according to smoking (*P* > .05) (Table 2).

**Table 2:** Cognitive function according to characteristics of male participants.

Characteristics	2014		*χ* ^2^	*P*	2016		*χ* ^2^	*P*	2018		*χ* ^2^	*P*
	Normal	Cognitive decline			Normal	Cognitive decline			Normal	Cognitive decline		
	N (%)	N (%)			N (%)	N (%)			N (%)	N (%)		
Age (years)												
50–59	472 (92.4)	39 (7.6)	139.353	<.001	350 (95.6)	16 (4.4)	232.032	<.001	203 (94.0)	13 (6.0)	248.194	<.001
60–69	658 (90.0)	73 (10.0)			660 (93.0)	50 (7.0)			637 (88.6)	82 (11.4)		
70–79	429 (76.6)	131 (23.4)			489 (77.7)	140 (22.3)			498 (75.2)	164 (24.8)		
≥ 80	104 (60.8)	67 (39.2)			155 (57.8)	113 (42.2)			188 (50.0)	188 (50.0)		
Education												
Elementary school	345 (68.5)	159 (31.5)	144.004	<.001	336 (67.1)	165 (32.9)	151.945	<.001	292 (58.3)	209 (41.7)	158.094	<.001
Middle school	294 (83.8)	57 (16.2)			294 (83.8)	57 (16.2)			267 (76.1)	84 (23.9)		
High school	680 (90.2)	74 (9.8)			683 (90.5)	72 (9.5)			639 (84.7)	115 (15.3)		
College and above	344 (94.5)	20 (5.5)			341 (93.2)	25 (6.8)			328 (89.4)	39 (10.6)		
Marital status												
With spouse	1555 (85.1)	272 (14.9)	12.668	<.001	1537 (84.5)	281 (15.5)	8.649	.003	1404 (78.3)	389 (21.7)	10.344	.001
Without spouse	108 (74.0)	38 (26.0)			117 (75.5)	38 (24.5)			122 (67.8)	58 (32.2)		
Household income												
< KRW 10 million	233 (67.0)	115 (33.0)	104.416	<.001	211 (64.5)	116 (35.5)	137.545	<.001	181 (56.6)	139 (43.4)	120.118	<.001
KRW 10–24.99 million	530 (84.7)	96 (15.3)			543 (81.7)	122 (18.3)			509 (75.1)	169 (24.9)		
≥ KRW 25 million	900 (90.1)	99 (9.9)			900 (91.7)	81 (8.3)			836 (85.7)	139 (14.3)		
Religion												
Religious	566 (86.0)	92 (14.0)	2.232	.135	557 (84.7)	101 (15.3)	0.488	.485	496 (81.2)	115 (18.8)	7.426	.006
Non-religious	1097 (83.4)	218 (16.6)			1097 (83.4)	218 (16.6)			1030 (75.6)	332 (24.4)		
Health condition												
Good	898 (90.0)	100 (10.0)	49.408	<.001	872 (90.6)	91 (9.4)	62.650	<.001	791 (86.9)	119 (13.1)	88.443	<.001
Poor	765 (78.5)	210 (21.5)			782 (77.4)	228 (22.6)			735 (69.1)	328 (30.9)		
Instrumental activities of daily living												
Restricted	121 (62.7)	72 (37.3)	75.321	<.001	160 (70.5)	67 (29.5)	33.714	<.001	105 (46.7)	120 (53.3)	136.395	<.001
Not restricted	1542 (86.6)	238 (13.4)			1494 (85.6)	252 (14.4)			1421 (81.3)	327 (18.7)		
Cerebrovascular disease												
Yes	47 (59.5)	32 (40.5)	38.201	<.001	69 (65.7)	36 (34.3)	26.857	<.001	66 (52.8)	59 (47.2)	45.880	<.001
No	1616 (85.3)	278 (14.7)			1585 (84.9)	283 (15.1)			1460 (79.0)	388 (21.0)		
Drinking												
Yes	1026 (86.7)	158 (13.3)	12.531	<.001	988 (87.2)	145 (12.8)	22.303	<.001	860 (82.1)	187 (17.9)	29.274	<.001
No	637 (80.7)	152 (19.3)			666 (79.3)	174 (20.7)			666 (71.9)	260 (28.1)		
Smoking												
Yes	479 (85.7)	80 (14.3)	1.156	.282	386 (88.7)	49 (11.3)	9.901	.002	298 (80.8)	71 (19.2)	3.020	.082
No	1184 (83.7)	230 (16.3)			1268 (82.4)	270 (17.6)			1228 (76.6)	376 (23.4)		
Workout												
Yes	704 (89.9)	79 (10.1)	30.991	<.001	737 (88.6)	95 (11.4)	23.949	<.001	640 (84.5)	117 (15.5)	36.338	<.001
No	959 (80.6)	231 (19.4)			917 (80.4)	224 (19.6)			886 (72.9)	330 (27.1)		
Neighborhood interaction												
Yes	1603 (85.3)	277 (14.7)	28.810	<.001	1580 (86.3)	251 (13.7)	113.578	<.001	1448 (80.5)	351 (19.5)	115.145	<.001
No	60 (64.5)	33 (35.5)			74 (52.1)	68 (47.9)			78 (44.8)	96 (55.2)		
Depression												
Yes	455 (72.8)	170 (27.2)	91.159	<.001	420 (69.9)	181 (30.1)	124.054	<.001	466 (63.2)	271 (36.8)	133.758	<.001
No	1208 (89.6)	140 (10.4)			1234 (89.9)	138 (10.1)			1060 (85.8)	176 (14.2)		
Life satisfaction												
Satisfied	1253 (88.4)	164 (11.6)	65.025	<.001	1301 (87.7)	183 (12.3)	65.029	<.001	1210 (82.4)	259 (17.6)	82.861	<.001
Not satisfied	410 (73.7)	146 (26.3)			353 (72.2)	136 (27.8)			316 (62.7)	188 (37.3)		
Economic activity												
Yes	1034 (88.8)	131 (11.2)	42.871	<.001	969 (91.2)	93 (8.8)	93.205	<.001	804 (84.9)	143 (15.1)	59.327	<.001
No	629 (77.8)	179 (22.2)			685 (75.2)	226 (24.8)			722 (70.4)	304 (29.6)		

KRW, Korean Republic won.

#### 3.2.2. Female participants

Analysis of 2014 data revealed significant differences in cognitive function according to age, education, marital status, household income, religion, health condition, IADL, cerebrovascular disease, drinking, workout, neighborhood interaction, depression, life satisfaction, and economic activity (*P < *.001).

Analysis of 2016 data revealed significant differences in cognitive function according to age, education, marital status, household income, religion, health condition, IADL, cerebrovascular disease, drinking, workout, neighborhood interaction, depression, life satisfaction, and economic activity (*P < *.05). Analysis of 2018 data revealed significant differences in cognitive function according to age, education, marital status, household income, religion, health condition, IADL, cerebrovascular disease, drinking, workout, neighborhood interaction, depression, life satisfaction, and economic activity (*P < *.01).

Notably, no smoking-related difference in cognitive function was observed in 2014, 2016, or 2018 (*P* > .05) (Table 3).

**Table 3:** Cognitive function according to characteristics of female participants.

Characteristics	2014		*χ* ^2^	*P*	2016		*χ* ^2^	*P*	2018		*χ* ^2^	*P*
	Normal	Cognitive decline			Normal	Cognitive decline			Normal	Cognitive decline		
	N (%)	N (%)			N (%)	N (%)			N (%)	N (%)		
Age (years)												
50–59	746 (93.5)	52 (6.5)	581.932	<.001	540 (92.6)	43 (7.4)	596.795	<.001	334 (91.8)	30 (8.2)	621.010	<.001
60–69	738 (79.4)	191 (20.6)			821 (82.8)	171 (17.2)			857 (83.8)	166 (16.2)		
70–79	449 (54.6)	374 (45.4)			480 (55.6)	384 (44.4)			520 (58.4)	371 (41.6)		
≥ 80	83 (29.0)	203 (71.0)			119(30.0)	278 (70.0)			160 (28.7)	398 (71.3)		
Education												
Elementary school	836 (56.0)	656 (44.0)	366.744	<.001	789 (53.0)	701 (47.0)	405.668	<.001	722 (48.5)	767 (51.5)	442.126	<.001
Middle school	408 (81.1)	95 (18.9)			399 (79.5)	103 (20.5)			396 (78.9)	106 (21.1)		
High school	641 (90.8)	65 (9.2)			645 (91.0)	64 (9.0)			629 (88.6)	81 (11.4)		
College and above	131 (97.0)	4 (3.0)			127 (94.1)	8 (5.9)			124 (91.9)	11 (8.1)		
Marital status												
With spouse	1577 (78.7)	427 (21.3)	192.295	<.001	1486 (77.8)	424 (22.2)	206.911	<.001	1376 (75.2)	454 (24.8)	195.276	<.001
Without spouse	439 (52.8)	393 (47.2)			474 (51.2)	452 (48.8)			495 (49.2)	511 (50.8)		
Household income												
< KRW 10 million	362 (48.7)	381 (51.3)	262.069	<.001	323 (45.0)	394 (55.0)	283.912	<.001	317 (44.8)	391 (55.2)	230.015	<.001
KRW 10–24.99 million	679 (74.4)	234 (25.6)			694 (71.9)	271 (28.1)			640 (65.8)	332 (34.2)		
≥ KRW 25 million	975 (82.6)	205 (17.4)			943 (81.7)	211 (18.3)			914 (79.1)	242 (20.9)		
Religion												
Religious	1084 (74.1)	378 (25.9)	13.738	<.001	989 (71.3)	399 (28.7)	5.844	.016	841 (69.0)	377 (31.0)	8.989	.003
Non-religious	932 (67.8)	442 (32.2)			971 (67.1)	477 (32.9)			1030 (63.7)	588 (36.3)		
Health condition												
Good	912 (84.8)	164 (15.2)	157.688	<.001	887 (83.0)	182 (17.0)	154.466	<.001	852 (82.0)	187 (18.0)	187.665	<.001
Poor	1104 (62.7)	656 (37.3)			1073 (60.7)	694 (39.3)			1019 (56.7)	778 (43.3)		
Instrumental activities of daily living												
Restricted	38 (24.7)	116 (75.3)	170.653	<.001	51 (24.4)	158 (75.6)	211.273	<.001	47 (18.7)	204 (81.3)	273.842	<.001
Not restricted	1978 (73.8)	704 (26.2)			1909 (72.7)	718 (27.3)			1824 (70.6)	761 (29.4)		
Cerebrovascular disease												
Yes	57 (51.4)	54 (48.6)	21.889	<.001	66 (50.4)	65 (49.6)	22.570	<.001	67 (45.9)	79 (54.1)	27.655	<.001
No	1959 (71.9)	766 (28.1)			1894 (70.0)	811 (30.0)			1804 (67.1)	886 (32.9)		
Drinking												
Yes	411 (80.9)	97 (19.1)	29.032	<.001	377 (80.4)	92 (19.6)	33.447	<.001	353 (80.8)	84 (19.2)	50.440	<.001
No	1605 (68.9)	723 (31.1)			1583 (66.9)	784 (33.1)			1518 (63.3)	881 (36.7)		
Smoking												
Yes	34 (64.2)	19 (35.8)	1.264	.261	29 (56.9)	22 (43.1)	3.650	.056	26 (61.9)	16 (38.1)	.314	.575
No	1982 (71.2)	801 (28.8)			1931 (69.3)	854 (30.7)			1845 (66.0)	949 (34.0)		
Workout												
Yes	702 (82.0)	154 (18.0)	71.176	<.001	730 (81.1)	170 (18.9)	88.927	<.001	678 (78.8)	182 (21.2)	90.987	<.001
No	1314 (66.4)	666 (33.6)			1230 (63.5)	706 (36.5)			1193 (60.4)	783 (39.6)		
Neighborhood interaction												
Yes	1942 (72.2)	748 (27.8)	31.169	<.001	1892 (72.3)	725 (27.7)	161.051	<.001	1789 (69.0)	805 (31.0)	121.359	<.001
No	74 (50.7)	72 (49.3)			68(31.1)	151 (68.9)			82 (33.9)	160 (66.1)		
Depression												
Yes	645 (56.4)	499 (43.6)	201.728	<.001	619 (55.1)	505 (44.9)	171.938	<.001	646 (51.8)	601 (48.2)	199.036	<.001
No	1371 (81.0)	321 (19.0)			1341 (78.3)	371 (21.7)			1225 (77.1)	364 (22.9)		
Life satisfaction												
Satisfied	1467 (78.4)	405 (21.6)	141.980	<.001	1517 (76.6)	463 (23.4)	173.070	<.001	1434 (74.3)	495 (25.7)	188.045	<.001
Not satisfied	549 (57.0)	415 (43.0)			443 (51.8)	413 (48.2)			437 (48.2)	470 (51.8)		
Economic activity												
Yes	744 (83.1)	151 (16.9)	92.266	<.001	668 (83.0)	137 (17.0)	101.296	<.001	595 (80.6)	143 (19.4)	95.378	<.001
No	1272 (65.5)	669 (34.5)			1292 (63.6)	739 (36.4)			1276 (60.8)	822 (39.2)		

KRW, Korean Republic won.

### 3.3. Effects of psychosocial characteristics on the participants’ cognitive function by year

#### 3.3.1. Male participants

A linear mixed model analysis was conducted to examine the effects of the male participants’ psychosocial characteristics on cognitive function and to test the differences in the change in cognitive function by year according to psychosocial characteristics. To examine the pure effects of psychosocial characteristics, we controlled for physical characteristics, including age, education, marital status, household income, religion, health condition, cerebrovascular disease, IADL, drinking, smoking, and workout.

The analysis revealed significant effects of depression (B = −0.589, *P = *.011) and time (B = −0.730, *P < *.001) on cognitive function. This implies that depression and advancing age have adverse effects on cognitive function.

Analysis of the interaction effects between psychosocial characteristics and year showed significant interactions between neighborhood interaction and year (B = 0.587, *P = *.003), depression and year (B = −0.231, *P = *.024), and economic activity and year (B = 0.201, *P = *.031). This can be interpreted to mean that neighborhood interaction and economic activity decreased the degree of cognitive decline over the years, while depression increased it.

**Figure 1.**
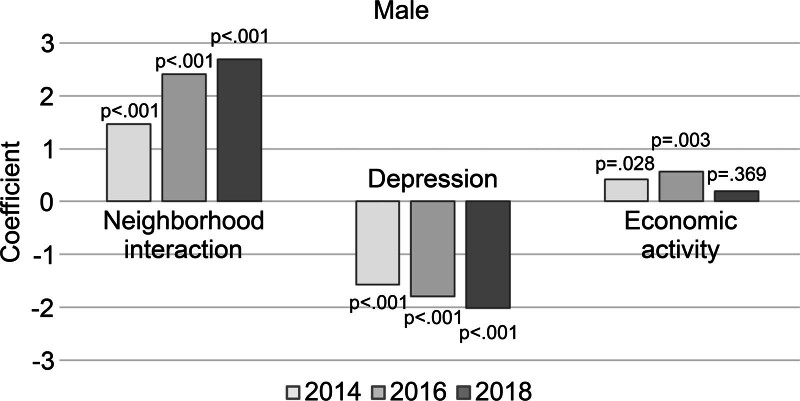
Plot of men’s cognitive function interaction of psychosocial characteristics and year.

However, among the control variables, significant interactions were found with age, education, household income, health condition, cerebrovascular disease, IADL, drinking, smoking, and workout. In older age, a lower education level (middle school graduation or lower vs college or higher), lower annual household income (≤10 million won vs ≥25 million won), and cerebrovascular disease were associated with lower cognitive function and good health condition with higher cognitive function. Restricted IADL significantly lowered cognitive function, and drinking and workout were associated with significantly higher cognitive function (Table 4, Figure 1). The finding that alcohol consumption improves cognitive function differs from what is commonly discussed in the literature. This is because the Aging Research Panel inquires only about the presence of alcohol consumption rather than alcohol use disorder, which has also been shown in other studies.^[35]^

**Table 4:** Mixed model analysis of the effect of men’s psychosocial characteristics on cognitive function.

Variables	B	SE	*t*	*P*	LLCI	ULCI
Age	−0.096	0.009	−10.317	<.001	−0.114	−0.078
Education						
Elementary school and under	−1.913	0.215	−8.879	<.001	−2.336	−1.491
Middle school	−0.724	0.220	−3.287	<.001	−1.156	−0.292
High school	−0.347	0.185	−1.877	.061	−0.709	0.016
College and above (ref)						
Marital status						
With spouse	0.295	0.219	1.346	.178	−0.135	0.725
Without spouse (ref)						
Household income						
<KRW 10 million	−0.533	0.173	−3.080	.002	−0.872	−0.194
KRW 10–24.99 million	0.147	0.120	1.231	.218	−0.087	0.382
≥KRW 25 million (ref)						
Religion						
Religious	0.101	0.100	1.004	.315	−0.096	0.298
Non-religious (ref)						
Health condition						
Good	0.295	0.219	1.346	.178	−0.135	0.725
Poor (ref)						
Cerebrovascular disease						
Yes	−2.046	0.264	−7.739	<.001	−2.564	−1.527
No (ref)						
Instrumental activities of daily living						
Restricted	−2.145	0.157	−13.621	<.001	−2.454	−1.836
Not restricted (ref)						
Drinking						
Yes	0.325	0.120	2.713	.007	0.090	0.560
No (ref)						
Smoking						
Yes	0.244	0.135	1.810	.070	−0.020	0.507
No (ref)						
Workout						
Yes	0.556	0.095	5.864	<.001	0.370	0.741
No (ref)						
Neighborhood interaction						
Yes	−0.042	0.463	−0.090	.928	−0.950	0.866
No (ref)						
Depression						
Yes	−0.589	0.231	−2.549	.011	−1.041	−0.136
No (ref)						
Life satisfaction						
Satisfied	0.224	0.244	0.916	.360	−0.255	0.702
Not satisfied (ref)						
Economic activity						
Yes	−0.314	0.224	−1.400	.162	−0.752	0.125
No (ref)						
Year	−0.730	0.204	−3.580	<.001	−1.129	−0.330
Neighborhood interaction × year	0.587	0.195	3.015	.003	0.205	0.969
Depression × year	−0.231	0.102	−2.258	.024	−0.432	−0.030
Life satisfaction × year	0.039	0.111	0.354	.724	−0.178	0.257
Economic activity × year	0.201	0.093	2.160	.031	0.019	0.384

KRW = Korean Republic won, LLCI = lower-limit confidence interval, SE = standard error, ULCI = upper-limit confidence interval.

#### 3.3.2. Female participants

A linear mixed model analysis was conducted to examine the effects of the female participants’ psychosocial characteristics on cognitive function and to evaluate the differences in the change in cognitive function by year according to psychosocial characteristics. To test the pure effects of psychosocial characteristics, we controlled for physical characteristics, including age, education, marital status, household income, religion, health condition, cerebrovascular disease, IADL, drinking, smoking, and workout. The analysis revealed significant effects of depression (B = −0.607, *P = *.002) and time (B = −0.441, *P = *.013) on cognitive function. This implies that depression and advancing age have adverse effects on cognitive function.

Analysis of the interaction effects between psychosocial characteristics and year revealed significant interactions between neighborhood interaction and year (B = 0.482, *P = *.004), and life satisfaction and year (B = 0.287, *P = *.002). This can be interpreted to mean that interacting with neighbors and being satisfied with life slowed down the degree of cognitive decline over the years.

**Figure 2.**
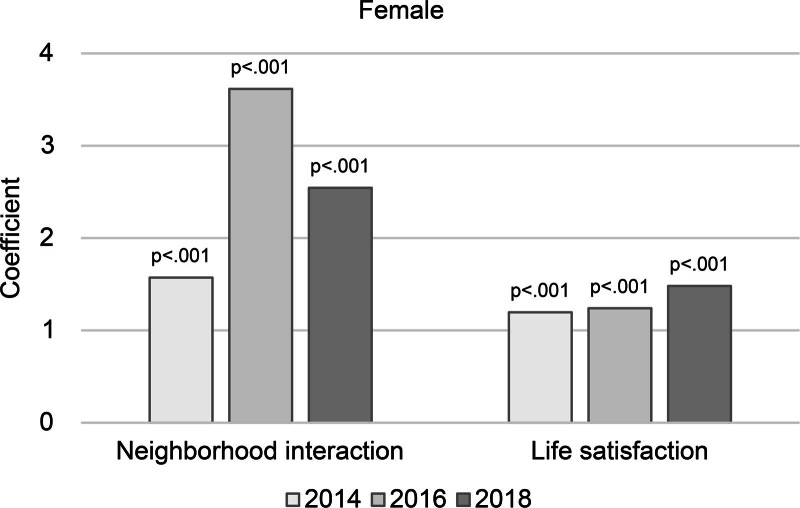
Plot of women’s cognitive function interaction of psychosocial characteristics and year.

However, among the control variables, significant interactions were found with age, education, marital status, household income, religion, health condition, cerebrovascular disease, IADL, drinking, and workout. In older age, a lower education level (middle school graduation or lower vs college or higher) and cerebrovascular disease were associated with lower cognitive function and good health condition with higher cognitive function. Restricted IADL significantly lowered cognitive function, whereas religion, drinking, and workout were associated with significantly higher cognitive function (Table 5, Figure 2).

**Table 5:** Mixed model analysis of the effect of women’s psychosocial characteristics on cognitive function.

Variables	B	SE	*t*	*P*	LLCI	ULCI
Age	−0.212	0.010	−21.987	<.001	−0.231	−0.193
Education						
Elementary school and under	−1.782	0.329	−5.412	<.001	−2.427	−1.136
Middle school	−0.666	0.337	−1.974	.048	−1.328	−0.005
High school	−0.413	0.324	−1.274	.203	−1.049	0.223
College and above (ref)						
Marital status						
With spouse	0.263	0.140	1.880	.060	−0.011	0.537
Without spouse (ref)						
Household income						
<KRW 10 million	−0.178	0.145	−1.229	.219	−0.463	0.106
KRW 10–24.99 million	0.180	0.113	1.590	.112	−0.042	0.403
≥KRW 25 million (ref)						
Religion						
Religious	0.229	0.086	2.656	.008	0.060	0.398
Non-religious (ref)						
Health condition						
Good	0.296	0.090	3.280	<.001	0.119	0.472
Poor (ref)						
Cerebrovascular disease						
Yes	−1.253	0.283	−4.422	<.001	−1.809	−0.698
No (ref)						
Instrumental activities of daily living						
Restricted	−1.253	0.283	−4.422	<.001	−1.809	−0.698
Not restricted (ref)						
Drinking						
Yes	0.360	0.158	2.272	.023	0.049	0.670
No (ref)						
Smoking						
Yes	0.072	0.420	0.173	.863	−0.750	0.895
No (ref)						
Workout						
Yes	0.503	0.090	5.600	<.001	0.327	0.679
No (ref)						
Neighborhood interaction						
Yes	0.416	0.397	1.048	.295	−0.362	1.194
No (ref)						
Depression						
Yes	−0.607	0.197	−3.077	<.001	−0.993	−0.220
No (ref)						
Life satisfaction						
Satisfied	−0.039	0.207	−0.188	.851	−0.444	0.366
Not satisfied (ref)						
Economic activity						
Yes	0.016	0.206	0.079	.937	−0.387	0.420
No (ref)						
Year	−0.441	0.178	−2.481	.013	−0.789	−0.092
Neighborhood interaction × year	0.482	0.169	2.858	.004	0.151	0.813
Depression × year	−0.089	0.088	−1.016	.310	−0.261	0.083
Life satisfaction × year	0.287	0.092	3.102	.002	0.105	0.468
Economic activity × year	0.039	0.088	0.446	.656	−0.134	0.212

KRW = Korean Republic won, LLCI = lower-limit confidence interval, SE = standard error, ULCI = upper-limit confidence interval.

## 4. Discussion

This study aimed to identify the factors associated with cognitive function in middle-aged and older adults (50 + men and women), using the relevant data for 4809 middle-aged and older adults whose complete data are available in all KLoSA panel data from 2014 to 2018, to contribute to improving the cognitive function of middle-aged and older adults. Therefore, changes in their cognitive function over the years were examined, and the effects of psychosocial characteristics (neighborhood interaction, depression, life satisfaction, and economic activity) on the cognitive function of middle-aged and older adults were evaluated.

Analysis of the cognitive function level of middle-aged and older adults by year and by sex led to the following findings: the normal cognitive function rates were 84.3%, 83.8%, and 77.3% in 2014, 2016, and 2018, respectively, in male participants and 71.1%, 69.1%, and 66.0% in 2014, 2016, and 2018, respectively, in female participants, indicating that normal cognitive decline progresses over the years, which is consistent with the research finding that cognitive function deteriorates with age.^[8]^ Although cognitive decline with age is a common phenomenon, some people have an earlier onset of cognitive decline than what is considered normal,^[36]^ and individual differences in cognitive decline in middle-aged and older adults, which are attributable to various causes, become increasingly pronounced with the lapse of time.^[37]^ Since interindividual differences in cognitive function are associated with individual differences in health status, lifestyle, socioeconomic status, and social relationships,^[38]^ cognitive function’s relationships with various psychosocial characteristics can have significant implications.

Analysis of the effects of the psychosocial characteristics on the cognitive function of middle-aged and older adults by sex revealed significant interactions of cognitive function with neighborhood and year in both male and female participants. This implies that for middle-aged and older active adults, interacting with neighbors can slow down the pace of cognitive decline over the years. Similarly, social activities and neighborhood interaction positively affected their cognitive function, which is consistent with the previous research findings that active social participation of middle-aged and older adults is positively correlated with their cognitive function, with intellectual stimulation from various social relationships contributing to maintaining cognitive function.^[39]^ Conversely, social isolation of middle-aged and older adults was found to lower their cognitive functional level.^[40]^ This is also in line with the research findings that interaction with others can reduce the progression of cognitive decline in middle-aged and older adults.^[27]^ Therefore, interaction with other members of society is necessary for middle-aged and older adults who communicate in complex interpersonal relationships. Such interactive communication activities provide opportunities to demonstrate cognitive capabilities, ultimately contributing to maintaining cognitive function in middle-aged and older adults.^[29]^ Neighborhood interaction refers to various leisure activities, such as volunteer and religious activities. Each social activity contributes to meeting the needs and enhancing the life satisfaction of middle-aged and older adults.^[41]^ Neighborhood interaction can play a crucial role in maintaining cognitive function by reducing depression and stress.^[29,41]^ These findings demonstrate that maintaining appropriate interactions with neighbors can help delay the onset or reduce the pace of cognitive decline over time.

Depression was also found to significantly impact cognitive function in both male and female participants. The higher the level of depression was, the more likely they were to have a lower cognitive function, which was more marked in all the participants over the years. It was also demonstrated in a previous study that the higher the depression score in middle-aged and older adults, the lower the cognitive function score, and that the depression score adversely affected the cognitive function score more markedly in the middle-aged and older adult group than in the adult group generally,^[42]^ indicating a vicious cycle of depression affecting cognitive function and cognitive decline affecting depression.^[24]^ Similarly, depression is recognized as a risk factor for cognitive dysfunction and has been reported as the biggest risk factor.^[43]^ This suggests that intervention against depressive symptoms in middle-aged and older adults prevents depression and contributes to maintaining cognitive function.

A significant correlation was observed between cognitive function and life satisfaction in the female participants rather than in the men. This variation may be primarily due to the sex difference in depression. It has been found that women are more vulnerable to depression than men among middle-aged and older adults,^[44]^ and they perceive old age earlier than men.^[45]^ Since female middle-aged and older adults are more depressive and more inclined to feel old than their male counterparts, they may be more sensitive to life satisfaction. In the study by Shim,^[46]^ no significant correlation was observed between work performance and quality of life, which is consistent with the finding of this study that quality of life and life satisfaction did not affect cognitive function in men. Conversely, cognitive function was found to positively influence life satisfaction,^[47]^ with cognitive decline acting as a factor lowering life satisfaction in older adults.^[48]^ This was consistent with the results of this study. Therefore, considering that the main focus of previous research has been on the effect of cognitive function on life satisfaction,^[49]^ it is necessary to understand the effect of life satisfaction on cognitive function. In addition, it is essential to explore strategies to prevent cognitive impairment or maintain cognitive function, starting from middle age.

A significant correlation was observed between cognitive function and economic activity in the male participants rather than in the women. This reflects the fact that women can engage in social activities even without their income in Korean society, such that a lack of economic activity does not affect their cognitive function. In a study by Hu et al,^[50]^ economic activity was a factor in lowering cognitive and emotional decline in middle-aged and older adults. Hu et al^[50]^ also found that economic activity is a factor mitigating the impairment of cognitive and affective functions in middle-aged and older adults, suggesting that economic activity in middle-aged and older adults is associated with their cognitive function. However, there is a gender difference in the effect of economic activity. For example, Yoon^[51]^ noted that men could lower their depression level through employment; however, employment can also increase the depression level in women. Based on the research finding that depression directly affects cognitive function^[52]^ this gender difference may be explained by the pathway through which employment affects depression, which leads to a positive result in men and a negative result in women. The responses to income and social activity (yes/no) do not necessarily coincide. Rather, the variable “neighborhood interaction” in this study is a more accurate indicator of social activity. This suggests that although the psychological satisfaction experienced through economic activity in men can add meaning to their lives and positively affect their cognitive function, economic activity is more likely to cause women depression than satisfaction, hardly affecting their cognitive function.

Other noteworthy findings evolve around control variables and cognitive function. For males, age, education level, household income, cerebrovascular disease, and instrumental daily living limitations had negative effects on cognitive function, while alcohol consumption and exercise had positive effects. For females, age, education level, cerebrovascular disease, and instrumental daily living limitations had negative effects on cognitive function, while religion, health condition, alcohol consumption, and exercise had positive effects. As previously mentioned, our finding that alcohol consumption is associated with improved cognitive function is attributed to the Aging Research Panel’s inquiring only about the presence of alcohol consumption, which is consistent with previous research.^[35]^ It is worth noting that the impact of cerebrovascular disease on cognitive function is similar to what has been reported previously, where patients with cerebrovascular disease experience difficulties in society.^[53]^

This study is significant because it confirmed the effects of psychosocial characteristics on cognitive function in middle-aged and older adults and tested the variables that showed interactions with changes over the years.

Based on the results of this study, the following proposals may be made:

First, the more actively the participants engaged in neighborhood interaction, the more likely they were, both male and female, to positively affect cognitive function. Therefore, since middle-aged and older adults can acquire cognitive stimuli from neighborhood interaction through new experiences away from their routine everyday life, it is crucial to set up measures to expand their opportunities to interact with neighbors. Additionally, it is necessary to encourage various programs for middle-aged and older adults in community-based senior colleges, welfare centers, senior well-being centers, and lifelong learning centers.

Second, depression was identified as a risk factor for cognitive function in both male and female middle-aged and older participants. Therefore, it is vital to prevent cognitive decline in middle-aged and older adults through continuous depressive management. Effective depression management can be achieved through various depression prevention programs developed and implemented based on the results of depression assessments through mental health psychological surveys of middle-aged and older adults in the community.

Third, life satisfaction was found to be a positive factor for cognitive function over the years in female middle-aged and older adults. Therefore, various measures should be established to improve life satisfaction in middle-aged and older women. Among many variables for enhancing their life satisfaction, they should engage in regular workouts and prevent physical function impairment. Therefore, it is necessary to develop workout programs that middle-aged and older women can participate in and to provide continuous education on maintaining physical function.

Fourth, economic activity was a positive factor for cognitive function in middle-aged and older male participants. Providing middle-aged and older men with the opportunity to work can increase their income and reduce the pace of their cognitive decline. Considering the rapid decrease in the economically active population in Korea due to its low fertility and increasingly aging population, the labor provided by middle-aged and older adults is expected to mitigate the negative effect of the workforce shortage. Socioeconomic activity plays a role in satisfying the basic needs of life and reduces the sense of loneliness by fostering a sense of participation and affiliation through social solidarity while simultaneously ensuring psychological and emotional stability.

Despite the significance of this study, there are several limitations. First, it should be emphasized that we used the KLoSA panel data. Due to the nature of secondary data, selecting items or questions necessary for testing specific variables is not possible, making it challenging to clarify causal relationships. This implies that measurement errors could not be controlled since the results were obtained from secondary data. Therefore, in follow-up studies, it is necessary to survey using a questionnaire containing items related to the research questions. Second, the Aging Research Panel survey data used in this study does not represent the entire elderly population and may be inaccurate to an extent since the survey relied on subjective responses. Third, this study utilized the 7th and 8th Aging Research Panel surveys, conducted at 2-year intervals, which may have excluded factors that affect cognitive decline over the long term. Therefore, it is suggested that future studies be planned as longitudinal studies with additional surveys to better understand the factual relationships.

Nevertheless, the significance of this study lies in confirming the influence of the socio-psychological characteristics of middle-aged and elderly individuals on cognitive function. Community health centers or social welfare agencies may utilize the results obtained in this study when implementing intervention programs for the middle-aged and elderly to help improve their cognitive function.

## Acknowledgments

We would like to thank Editage (www.editage.com) for English language editing and journal submission support. The authors have authorized the submission of this manuscript through Editage.

## Author contributions

**Formal analysis:** Ji-Young Park, Hye-Sun Jung.

**Investigation:** Ji-Young Park.

**Methodology:** Hye-Sun Jung.

**Project administration:** Hye-Sun Jung.

**Software:** Ji-Young Park.

**Supervision:** Hye-Sun Jung.

**Validation:** Ji-Young Park, Hye-Sun Jung.

**Visualization:** Ji-Young Park, Hye-Sun Jung.

**Writing – original draft:** Ji-Young Park, Hye-Sun Jung.

**Writing – review & editing:** Ji-Young Park, Hye-Sun Jung.
